# Zinc Polyaleuritate Ionomer Coatings as a Sustainable, Alternative Technology for Bisphenol A-Free Metal Packaging

**DOI:** 10.1021/acssuschemeng.1c04815

**Published:** 2021-11-03

**Authors:** Davide Morselli, Pietro Cataldi, Uttam Chandra Paul, Luca Ceseracciu, Jose Jesus Benitez, Alice Scarpellini, Susana Guzman-Puyol, Antonio Heredia, Paola Valentini, Pier Paolo Pompa, David Marrero-López, Athanassia Athanassiou, José Alejandro Heredia-Guerrero

**Affiliations:** † Smart Materials, Istituto Italiano di Tecnologia, Via Morego 30, 16163 Genova, Italy; ‡ Department of Civil, Chemical, Environmental and Materials Engineering (DICAM), Università di Bologna, Via Terracini 28, 40131 Bologna, Italy; § Center for Nano Science and Technology@PoliMi, Istituto Italiano di Tecnologia, Via G. Pascoli 70/3, 20133 Milan, Italy; ∥ Materials Characterization Facility, Istituto Italiano di Tecnologia, Via Morego 30, 16163 Genova, Italy; ⊥ Instituto de Ciencia de Materiales de Sevilla, Centro Mixto CSIC-Universidad de Sevilla, Americo Vespucio 49, Isla de la Cartuja, Sevilla 41092, Spain; # Electron Microscopy Facility, Istituto Italiano di Tecnologia, Via Morego, 30, Genova 16163, Italy; ∇ Instituto de Hortofruticultura Subtropical y Mediterránea “La Mayora”, Universidad de Málaga-Consejo Superior de Investigaciones Científicas (IHSM, UMA-CSIC), Bulevar Louis Pasteur, 49, 29010 Málaga, Spain; ○ Departamento de Biología Molecular y Bioquímica, Facultad de Ciencias, Instituto de Hortofruticultura Subtropical y Mediterránea “La Mayora”, Universidad de Málaga-Consejo Superior de Investigaciones Científicas (IHSM, UMA-CSIC), E-29071 Málaga, Spain; ◆ Nanobiointeractions & Nanodiagnostic, Istituto Italiano di Tecnologia, Via Morego 30, 16163 Genova, Italy; ¶ Dpto. de Física Aplicada I, Universidad de Málaga, 29071 Málaga, Spain

**Keywords:** sustainable lacquer, can coating, polyaleuritate, ZnO nanoparticles, metal packaging, bisphenol A-free

## Abstract

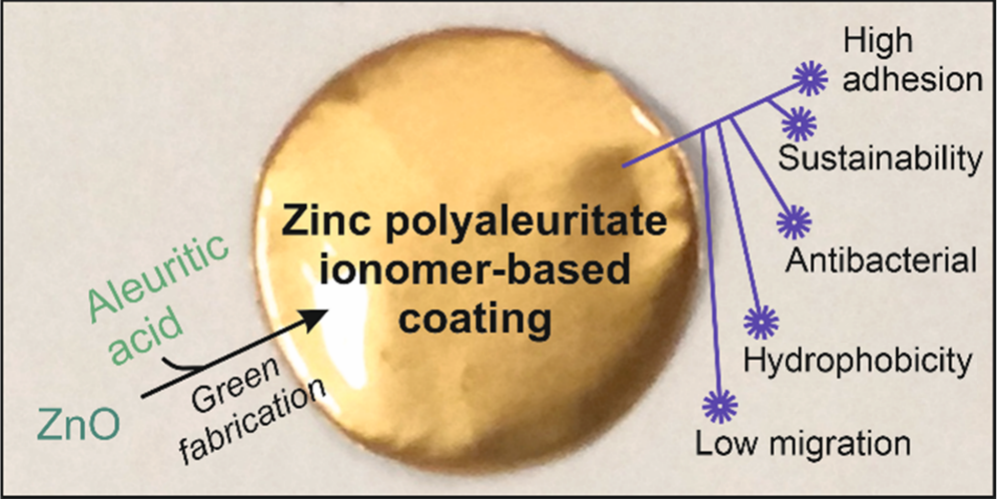

Sustainable coatings for metal food packaging were prepared from ZnO nanoparticles (obtained by the thermal decomposition of zinc acetate) and a naturally occurring polyhydroxylated fatty acid named aleuritic (or 9,10,16-trihydroxyhexadecanoic) acid. Both components reacted, originating under specific conditions zinc polyaleuritate ionomers. The polymerization of aleuritic acid into polyaleuritate by a solvent-free, melt polycondensation reaction was investigated at different times (15, 30, 45, and 60 min), temperatures (140, 160, 180, and 200 °C), and proportions of zinc oxide and aleuritic acid (0:100, 5:95, 10:90, and 50:50, w/w). Kinetic rate constants calculated by infrared spectroscopy decreased with the amount of Zn due to the consumption of reactive carboxyl groups, while the activation energy of the polymerization decreased as a consequence of the catalyst effect of the metal. The adhesion and hardness of coatings were determined from scratch tests, obtaining values similar to robust polymers with high adherence. Water contact angles were typical of hydrophobic materials with values ≥94°. Both mechanical properties and wettability were better than those of bisphenol A (BPA)-based resins and most likely are related to the low migration values determined using a hydrophilic food simulant. The presence of zinc provided a certain degree of antibacterial properties. The performance of the coatings against corrosion was studied by electrochemical impedance spectroscopy at different immersion times in an aqueous solution of NaCl. Considering the features of these biobased lacquers, they can be potential materials for bisphenol A-free metal packaging.

## Abstract 2

Sustainable zinc polyaleuritate ionomer-based coatings are fabricated by green manufacturing and presented as an alternative technology for BPA-free metal packaging.

## Introduction

Metal packaging represents globally ∼15% of the packaging, being an important business that is estimated to reach ∼58 billion US dollars in 2024.^1^ Leading manufacturers of metal cans are North America and Europe, with market shares of 32 and 30%, respectively, of a global production of ∼430 billion cans in 2020.^1^ Metal packaging forms a barrier against light, oxygen, and pathogens that protects canned food from the environment and increases its shelf-life, keeping food quality for long times. Other valuable properties are recyclability, processability in a wide range of shapes and sizes, and easy branding with many decorative options.^2^

Main metallic substrates used for packaging are tin plate, tin-free steel, stainless steel, and, mostly, aluminum.^3^ However, metals can react with food, usually initiating corrosion and releasing toxic substances. For example, aluminum cans are covered by a thin Al_2_O_3_ passivation layer that is spontaneously formed when Al is in contact with air or water. This layer is mechanically robust and chemically inert, although its solubility increases at low and high pH and high NaCl concentrations. In such conditions, corrosion occurs and food preservation is unsatisfactory.^4,5^ To avoid such metal–food interactions, metal cans are coated with lacquers or resins. Main lacquers for metal cans are acrylic, epoxy, phenolic, polyester, and vinyl resins as well as oleoresins.^6^ Acrylic resins are mainly prepared from ethylacrylate polymerization. They show high heat resistance and good color retention, although are brittle and give odor and typically are blended with epoxy resins. Phenolic lacquers result from the condensation of formaldehyde with different types of phenols. They have good chemical resistance but present high curing times and brittleness. Similar to acrylics, they are usually combined with epoxies. Polyester resins for cans are prepared by condensation of carboxyl groups with alcohols or epoxies and subsequent cross-linking. Their flexibility can be easily tuned and does not impart flavor or odor to foods. However, they fail with chemically aggressive foods and their poor corrosion resistance. Vinyl lacquers are synthesized by polymerization of vinyl monomers, mainly vinyl chloride and acetate, typically with other co-monomers such as maleic acid or anhydride. They display excellent flexibility and good chemical resistance, although are heat-sensitive and cannot be used in steam sterilization processes. Oleoresins are fabricated by a combination of natural gums and resins with drying oils (mainly tung and linseed oils). They are low-cost and show good resistance against fruits acids. Their main weaknesses are related to poor corrosion resistance and low adhesion to the metal substrates. Epoxy resins stand out in all technologic performances such as corrosion resistance, ease of fabrication, simplicity of coating, organoleptic features, appearance, and low cost.^7^ Epoxy resins, with a market share of ∼95% for metal packaging, result from the chemical reaction between bisphenol A (BPA) and epichlorohydrin in basic media.^8^ Nevertheless, BPA is related to several sustainability and human health issues. The large-scale production of BPA consists of the reaction in the bulk of acetone and phenol initiated by thiols and catalyzed by strong acids.^9^ In addition, the migration of BPA to canned food is associated with cardiovascular disease, cancer, and diabetes, among other diseases, and induces reproductive anomalies and developmental effects due to the BPA chemical structure, which is similar to estrogens.^10−13^

Many important chemical companies (*e.g*., AkzoNobel, Eastman Chemical, Dow Chemical, *etc*.) are developing safe and cheap alternatives to BPA-based resins with low environmental impact, although there are no candidates with good performances in all technical requirements.^7,14^ In this scenario, the production of biodegradable, safe, and innocuous materials inspired by nature with properties similar to those of BPA resins can be considered a potential alternative. Aleuritic (9,10,16-trihidroxyhexadecanoic) acid is presented here as a promising candidate. It is the main component (∼35 wt %) of shellac, a natural lac resin mainly produced in India.^15,16^ This polyhydroxylated fatty acid is currently used in cosmetic formulations and as a food additive and coating material for tablets and capsules.^17^ The polymerization of this polyhydroxylated fatty acid into polyaleuritate by a solvent-free, noncatalyzed melt polycondensation has been optimized.^18−23^ This biobased polyester is a hydrophobic, waterproof, ductile, insoluble, infusible, and cross-linked polymer that fungi and other microorganisms can biodegrade.

Zinc oxide (ZnO) is generally recognized as a safe (GRAS) material by the U.S. Food and Drug Administration (FDA).^24^ A similar behavior is proposed by the European Food Safety Authority.^25,26^ It is used as an additive of food packaging materials due to its antimicrobial features and its improvement in the mechanical, barrier, and thermal properties.^27^ However, the migration of ZnO nanoparticles to food can be a health issue for consumers. In this sense, European legislation has imposed a specific migration limit from 5 to 25 mg zinc per kg food for food contact items.^28^ ZnO is also a common additive of rubbers since it is used as a vulcanizing activator.^29^ There are many methodologies to synthesize ZnO (*e.g*., pyrometallurgical or hydrometallurgical synthesis, precipitation from aqueous solutions of zinc salts, solvent extraction and pyrolysis of zinc nitrate, gas-phase synthesis, *etc*.).^29^ However, the thermally activated conversion of the zinc acetate precursor is preferred due to its simplicity, good efficiency, low energetic requirements, and easy *in situ* formation of ZnO nanoparticles in polymer matrices.^30−33^ ZnO is amphoteric and can react with fatty acids to form the corresponding zinc carboxylates, usually named zinc soaps, and with applications mainly in paints.^34,35^ ZnO can also react with −COOH or anhydride groups present in polymers to form zinc carboxylates named ionomers.^36,37^ These ionomers have found application as dental materials.^38^

In this work, we report the fabrication process of green, zinc polyaleuritate ionomer-based coatings for metal packaging of foodstuffs as potential substitutes for BPA lacquers. For this, a scalable procedure that consists of the formation of ZnO nanoparticles by thermal decomposition from zinc acetate precursors and successive polymerization of aleuritic acid by melt polycondensation is proposed. The synthesis of ZnO nanoparticles on aluminum substrates was finely characterized, while the reaction between the fatty acid and the metal oxide and the kinetics of aleuritic acid polymerization in the presence of different ZnO contents was determined by infrared spectroscopy. The morphology and mechanical and antibacterial properties of the coatings were investigated. Finally, the overall and specific migrations were assessed.

## Experimental Section

### Materials

Aleuritic (9,10,16-trihydroxyhexadecanoic) acid (98% purity) was purchased from TCl Europe. Zinc acetate dihydrate (Zn(OAc)_2_·2H_2_O, 99.999% purity), methanol (MeOH, LC-MS Chromasolv), hydrochloric acid (HCl, 37%, ACS reagent), and nitric acid (HNO_3_, 70%, ACS reagent) were purchased from Sigma Aldrich. All reported chemicals were high-purity reagents and used as received without any further purification. An aluminum foil, used as a substrate (25 mm × 50 mm and 30 μm thick), was purchased from RS Components. For mechanical characterization, a thicker (∼1 mm) aluminum substrate was used.

### Coating Preparation

**Figure 1:**
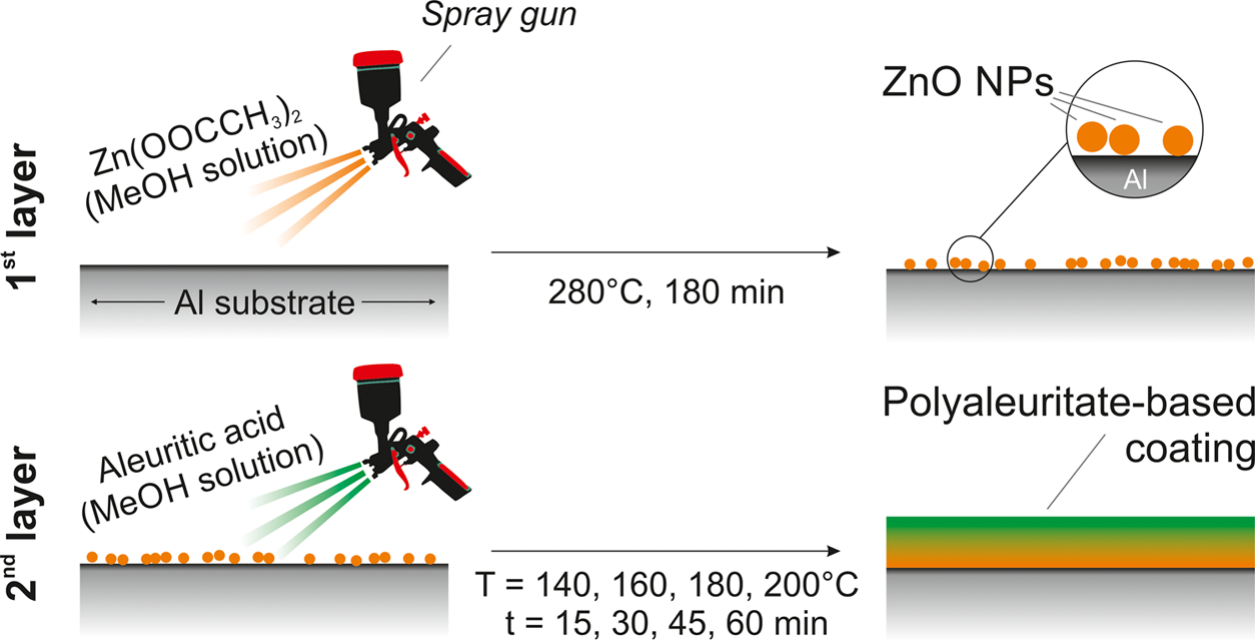
Schematic representation of the employed two-step procedure for the coating preparation.

A Zn(OAc)_2_ solution (0.01 g/mL, 2.6 mL) in methanol was sprayed on top of Al substrates employing a Paasche air brusher (0.73 mm nozzle, model VL siphon feed) positioned at 15 cm from the substrate and applying an air pressure of 1.5 bar.^39,40^ After the spray coating process, annealing was performed in an oven (Air Concept, Firlambo) at 280 °C for 180 min to induce the thermal degradation of Zn(OAc)_2_ and obtain a homogeneous coating of ZnO nanoparticles.^30,31^ The as-obtained ZnO-modified Al substrates were spray-coated a second time with 2.6 mL of a solution with different concentrations of aleuritic acid (*i.e*., 0.1, 0.05, and 0.01 g/mL that corresponded to ZnO/aleuritic acid weight ratios of 5:95, 10:90, and 50:50 and were labeled as AZ-95, AZ-90, and AZ-50, respectively) dissolved in methanol. The spray was performed with the same setup and conditions described above for the Zn(OAc)_2_ methanol solution, as reported in Figure 1. A control sample (AZ-100) without a previous ZnO layer was prepared by the spray of an aleuritic acid methanol solution at 0.1 g/mL directly on the aluminum substrate. A second thermal treatment was necessary to polymerize aleuritic acid into polyaleuritate. To characterize the kinetics of the polymerization process, different temperatures (140, 160, 180, and 200 °C) and times (15, 30, 45, and 60 min) were applied. Lower times and temperatures showed no polymerization of the polyhydroxylated fatty acid. On the other hand, no better results were achieved for longer times and higher temperatures.

### Morphological Characterization

The morphology and dimensions of the primary ZnO nanoparticles were investigated with a JEOL JEM-1011 transmission electron microscope (TEM) equipped with a tungsten thermionic electron source and integrated selected area electron diffraction (SAED) pattern analysis, operating at 100 kV. A specimen (10 mm × 10 mm) of the ZnO-coated Al substrate was dipped in 2 mL of methanol and left shaking overnight. Five microliters of the obtained diluted suspension was placed on a copper grid (300 Mesh Cu Carbon only) and dried at room temperature. TEM images were further analyzed by FIJI open-source software to evaluate the particle size distribution (∼100 NPs were measured). In addition, the topography of the ZnO nanoparticles on the aluminum substrate was characterized by atomic force microscopy (AFM). AFM images were acquired using a Nanotec microscope (Nanotec, Spain) in a low amplitude dynamic mode. Levers used were Nanosensors PPP-NCH (NanoWorld AG, Switzerland) with a tip radius curvature of less than 10 nm and a resonance frequency of 295 kHz (29 N/m force constant). WSxM software was used for the correction and analysis of the images.^41^

The morphology of the coatings was characterized using a JEOL JSM 7500FA high-resolution scanning electron microscope (HR-SEM) equipped with a cold field emission gun, applying an accelerating voltage of 15 kV and a chamber pressure of 9.6 × 10^–5^ Pa. The cross-sections were prepared by fracturing the specimens in liquid nitrogen to minimize the plastic deformation of the films and preserve their multilayer structure.

### Structural Characterization

X-ray diffraction (XRD) measurements were performed with a PANalytical Empyrean X-ray diffractometer equipped with a 1.8 kW Cu Kα ceramic X-ray tube (1.5418 Å) and a PIXcel^3D^ 2 mm × 2 mm area detector, operating at 45 kV and 40 mA. The reflections were collected at room temperature using a parallel-beam geometry and symmetric reflection mode, in a 2θ range of 15–70° with a step time of 450 s and a step size of 0.1°, repeating the measurement four times to reduce the signal noise. The XRD results were analyzed using HighScore 4.1 software (PANalytical).

### Chemical Characterization

Infrared spectra were obtained with a single-reflection attenuated total reflection (ATR) accessory (MIRacle ATR, PIKE Technologies) coupled to a Fourier transform infrared (FTIR) spectrometer (Equinox 70 FT-IR, Bruker). All spectra were recorded in the range from 3800 to 600 cm^–1^ with a resolution of 4 cm^–1^, accumulating 128 scans. To assess the homogeneity of chemical composition, ATR-FTIR spectra were repeated in three different areas. Band fitting of C=O components (1800–1500 cm^–1^) was carried out using PeakFit 4.11 software. Wavenumber positions of the different components (*i.e*., free ester groups, esters interacting by H bonds, carboxyl groups, and carboxylates) were determined by calculation of the second-order derivative. Deconvolution was performed using a Gaussian shape with an amplitude threshold of 3%. A nonlinear least-square method was employed to reduce the differences between the calculated spectra and the original one.

### Mechanical Characterization

The resistance of the films to deformation or removal by accidental localized contact was evaluated by scratch tests on an Anton Paar Micro Combi scratch/indenter. In particular, tests with a progressively increased load were performed to evaluate the coating adhesion to the substrate, while tests at constant load determined the scratch hardness, *i.e*., the resistance to permanent deformation upon sliding of a sharp tip. Scratch tests were performed with a conical diamond Rockwell tip (*R* = 0.1 mm) sliding on the surface with a progressively increasing load, from 0.03 to 1 N. Additional tests were conducted with a higher maximum load, namely, 2 N, when necessary to induce damage. The tip was displaced with a rate of 2 mm/min for a length of 2 mm. After each scratch, optical observation with a built-in microscope defined the critical loads for the onset of damage. Additional tests were performed under the constant load *P* = 1 N, for a length of 5 mm. From these, the scratch hardness *H*_s_ was calculated following the indication of the ASTM G171-03 standard: the scratch width *w* was measured in three points along with the scratch (1, 2.5, and 4 mm), and the scratch hardness was calculated as follows
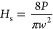
1Both types of tests were replicated three times for each material.

### Electrochemical Characterization

The anticorrosion performance of the coatings was studied by electrochemical impedance spectroscopy (EIS) in a two-electrode cell using a homemade electrochemical setup, in which the coating metal substrate was the working electrode and a Pt mesh served as the counter electrode. The impedance spectra were collected with a Solartron 1260 frequency response analyzer in the range of 5 mHz to 1 MHz (8 points per decade), using an ac amplitude of 50 mV at the open-circuit voltage. The coating surface with an active surface area of 1 cm^2^ was exposed to an aqueous solution of 1 wt % NaCl. The impedance spectra were acquired in a time interval of 2–5 h during an overall stability test of 100 h. An unprotected aluminum substrate was also studied for comparison purpose. The spectra were analyzed by equivalent circuit models with ZView software (Scribner Associates) to determine the resistance and capacitance of the different processes occurring at the metal/coating interface.

### Wettability

Static water contact angle measurements were performed using the sessile drop method using a DataPhysics OCAH 200 contact angle goniometer equipped with a CCD camera and image processing software operating under laboratory conditions (temperature of 22–25 °C and relative humidity of 50–60%). For the characterization, 1 μL droplets of Milli-Q water were used. Up to 10 contact angle measurements were carried out on every sample at random locations, and their average values were reported.

### Antibacterial Tests

For the antibacterial tests, *Escherichia coli* ATCC 25404 was used. The samples and controls tested were AZ-100, AZ-95, AZ- 90, AZ-50, a plain aluminum foil, and the plastic well surface (without the aluminum foil); 1 cm^2^ of each sample was put in a sterile multiwell plate and 80 μL of bacterial culture was placed at the center. Three different starting numbers of bacteria colony forming units (CFU) were tested: 4 × 10^6^, 8 × 10^6^, and 1.6 × 10^7^ CFU. The plates were incubated at room temperature and protected from light. After 8 h, an 80 μL drop was collected and placed in a cuvette. The samples were washed with 120 μL of fresh medium to collect all of the bacteria that could be deposited on the film. The washing medium was added to the measurement cuvette, the optical density at 600 nm (OD_600_) was measured, and the corresponding CFU were calculated.

### Overall Migration Analysis

The overall migration of packaging materials was done using an ethanol/water (10% v/v) aqueous food simulant, as described in the European Standard 1186-1:2002 “Materials and articles in contact with foodstuffs. Plastics. Part 1: Guide to the selection of conditions and test methods for overall migration”. A circular strip with a diameter of 19.3 mm of each test sample was immersed in 10 mL of the food simulant. The glass vials were covered with parafilm to avoid the evaporation of the simulant during the contact period and kept in an oven at 40 ± 0.5 °C for 10 days. After this period, the samples were removed and 5 mL of each simulant was placed in a preweighed glass Petri dish and the simulant was evaporated at room temperature. The glass Petri dish containing the residue of evaporation was kept in an oven at 105 ± 1.0 °C for 2 h followed by 4 h in a desiccator and then weighed. An analytical balance with an accuracy of 0.001 mg was used to weigh the samples. Blank samples were simultaneously run and corrected migration values were calculated. The mass determination of the residue was done by subtracting the original stable mass of the glass Petri dish from the stable mass of the glass Petri dish and the residue. The overall migration *M*, as milligrams of residue per square decimeter of the surface of the sample that is intended to come into contact with foodstuffs, was calculated for each test specimen using the following formula
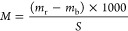
2where *m*_r_ is the mass of the residue from the test specimen after evaporation of the simulant in which it had been immersed (in grams), *m*_b_ is the mass of the residue from the blank simulant (in grams), and *S* is the surface area of the test specimen intended to come into contact with foodstuff (in square decimeters).

For each sample, three measurements were performed, the final migration value was averaged, and the standard deviation was calculated.

### Determination of the Specific Migration

The concentration of zinc oxide was determined by elemental analysis using an ICP-AES (inductive coupled plasma-atomic emission) spectrometer (iCAP 6500, Thermo). The collected food simulant during the overall migration analysis experiments was digested in *aqua regia* (a mixture of concentrated nitric and hydrochloric acids at a molar ratio of 1:3). Specifically, 250 μL of the collected liquid sample was mixed with 2.5 mL of *aqua regia* and left overnight for the complete digestion of zinc ions. Then, the solution was further diluted with Milli-Q water up to 25 mL and filtered through 0.45 μm poly(tetrafluoroethylene) (PTFE) filters prior to zinc ion analysis. The polyaleuritate content was then estimated by subtracting the zinc content from the overall migration values.

## Results and Discussion

### Zinc Oxide Nanoparticles are Formed on the Al Substrate from Zinc Acetate by Thermal Treatment

**Figure 2:**
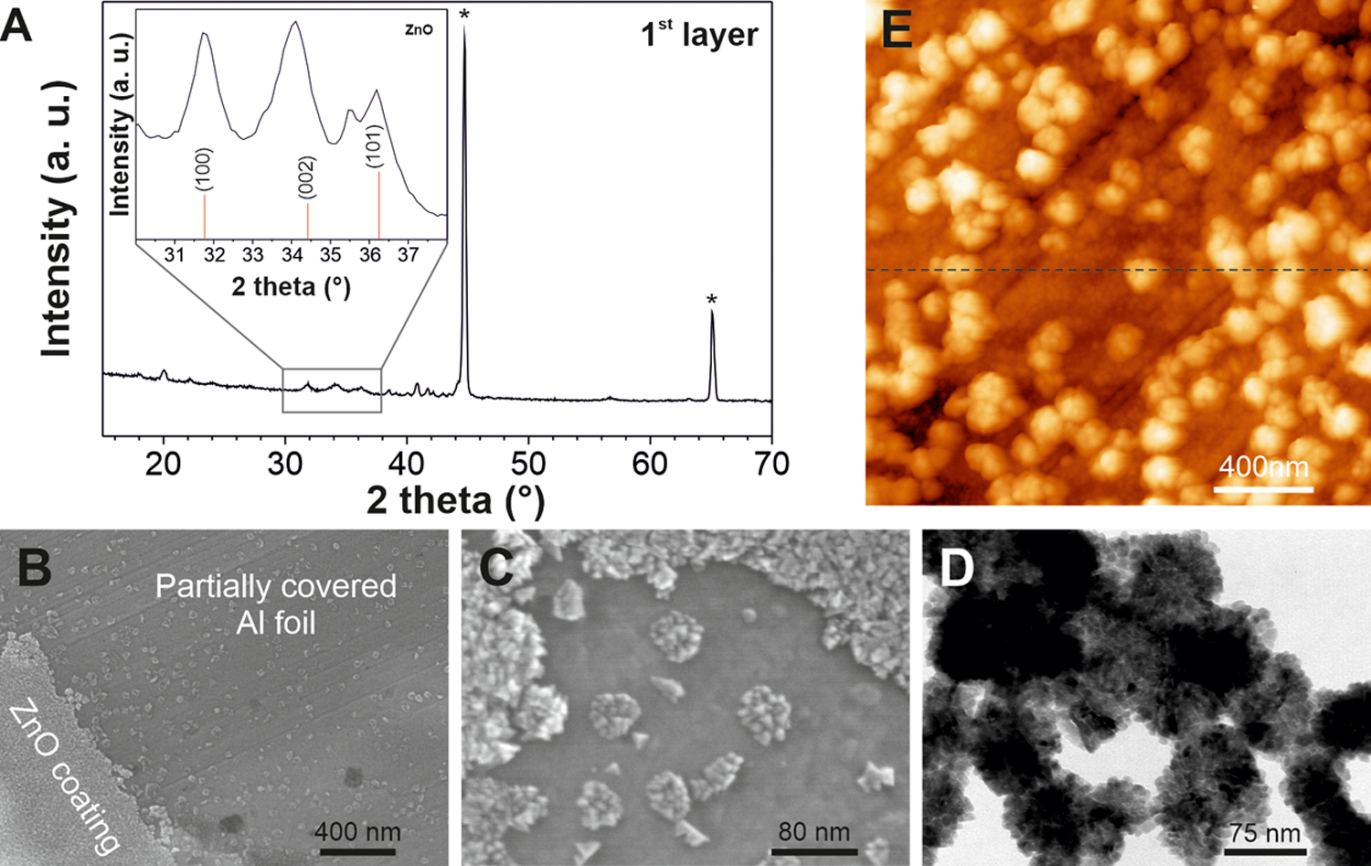
(A) XRD diffractogram of the ZnO-modified Al substrate after thermal treatment at 280 °C for 3 h. The inset shows the range between 30 and 38° and the associated ZnO database pattern (JCPDS file 01-079-0206, in red). (B, C) HR-SEM top-view micrographs of the ZnO-modified Al substrate after the thermal treatment at different magnifications. (D) TEM image of the branched ZnO NPs. (E) AFM topography of the ZnO nanoparticles covering the aluminum substrate. The corresponding height profile of the dashed line is shown in Figure S1D.

The formation of ZnO from the zinc acetate precursor was determined by XRD (Figure 2A). The most intense peaks at 44 and 65° were associated with the aluminum substrate, as demonstrated by the diffractogram of the bare substrate reported in Figure S1A. The reflection at 33° (Figure S1A) ascribable to Zn(OAc)_2_ (JCPDS file 00-056-0569) disappeared after the thermal treatment, proving that the thermal decomposition of the precursor occurred. The reflection peaks due to the formation of ZnO NPs arose from the background after the annealing (inset of Figure 2A), revealing that the prepared NPs are characterized by a hexagonal crystalline structure, typical of a zincite phase (JCPDS file 01-079-0206). The morphology and size of the ZnO NPs and the multilayer structure were characterized through a combination of HR-SEM, AFM, and TEM techniques (Figure 2B–E). The HR-SEM images (Figure 2B) show the ZnO NPs before the spray deposition of the aleuritic acid layer on top of the aluminum substrate. To distinguish better the ZnO layer, such image displays a border between the ZnO coating and the Al substrate (the typical situation with a complete ZnO coating of the Al substrate is presented in Figure S1B). In this region, it is possible to observe both the ZnO layer (on the left) and easily identifiable isolated NPs (Figure 2C). In Figure 2D, a representative TEM micrograph shows the branched morphology of the synthesized NPs, which is comparable to that previously observed for ZnO NPs *in situ* grown in PMMA fibers.^30^ As observed, small ZnO NPs aggregate forming larger clusters with diameters of 75–100 nm. The particle size distribution of the zinc oxide nanoparticles, as reported in Figure S1C, was investigated by analyzing several TEM images, showing that approximately 70% of the NPs have dimensions that ranged from 4 to 11 nm. SAED measurement (Figure S1D) performed on the observed NPs is in good agreement with the XRD results, confirming the formation of ZnO NPs. Similar to TEM images, the clusters of ZnO NPs on the aluminum foil were also easily observed by AFM (Figure 2E). The dimensions of ZnO clusters, estimated by height profiles (an example is shown in Figure S1E), were estimated to be 40–50 nm in height and 150–175 nm in width.

### ZnO Reacts with Aleuritic Acid and Catalyzes the Polymerization

**Figure 3:**
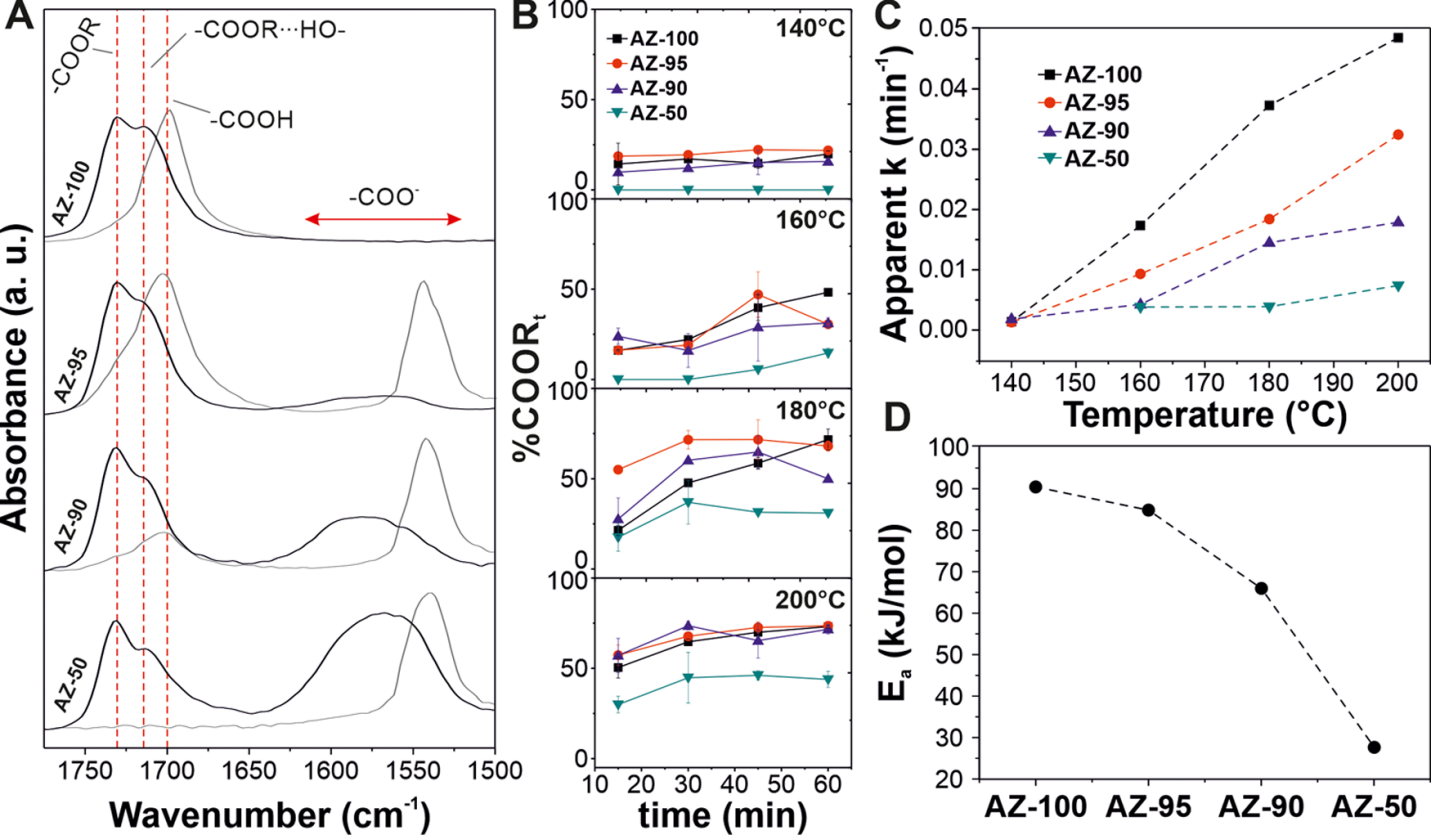
(A) C=O stretching mode of AZ-100, AZ-95, AZ-90, and AZ-50 coatings before (gray line) and after (black line) thermal treatment at 200 °C for 1 h. The different chemical environments of C=O functional groups have been indicated. (B) Total ester percentage as a function of reaction time at 140, 160, 180, and 200 °C. (C) Variation of the calculated apparent kinetic constants with the reaction temperature for AZ-100, AZ-95, AZ-90, and AZ-50 coatings. (D) Energies of activation of the melt polycondensation of AZ-100, AZ-95, AZ-90, and AZ-50.

AZ coatings before and after the thermal treatment to induce aleuritic acid polymerization were chemically characterized by ATR-FTIR spectroscopy (Figure 3). Figure 3A shows the spectral region from 1775 to 1500 cm^–1^ where main chemical modifications occurred. Before the thermal treatment, AZ-100 exhibited a single peak at 1699 cm^–1^, which was attributed to the C=O stretching mode of aliphatic carboxyl groups, typical of aleuritic acid.^42^ However, a new sharp peak at ∼1540 cm^–1^ appeared in zinc-containing samples. This band was related to the antisymmetric −COO^–^ stretching mode of zinc carboxylates that result from the acid–base reaction between −COOH groups of fatty acids and ZnO^43^
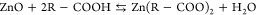
3The intensity of this peak depended directly on the relative amount of zinc, with higher intensities for zinc-richer coatings. Thus, the ratio *I*_COOH_/*I*_COO_^_–_^ was ∼1.2, ∼0.3, and 0 for AZ-95, AZ-90, and AZ-50, respectively. The position and shape of this band are indicative of crystalline zinc soaps of saturated fatty acids where long-chain packing is optimized.^43,44^ After the thermal treatment, the peak at 1699 cm^–1^ shifted to higher wavenumbers typical of the C=O stretching mode of free esters (∼1730 cm^–1^) and esters interacting by H bonds (∼1714 cm^–1^), revealing the polymerization of aleuritic acid into polyaleuritate. In addition, the antisymmetric −COO^–^ stretching mode shifted to ∼1575 cm^–1^ and the peak became broader. This situation has been ascribed to zinc ions interacting with carboxylate groups in polymer matrices forming ionomer structures.^44^ The contribution of the total ester (the sum of free and interacting by H bond esters), carboxyl, and carboxylate groups to the C=O stretching mode was calculated by deconvolution (Figures 3B and S2). As observed in Figure 3B, the percentage of total esters increased with the reaction time and temperature, reaching the best results for the samples prepared at 200 °C for 60 min: ∼73% for AZ-100, ∼74% for AZ-95, ∼72% for AZ-90, and ∼44% for AZ-50. The %COOR_t_ was lower, as the zinc amount was higher, which can be associated with the loss of carboxyl groups transformed in zinc carboxylates, decreasing the number of collisions between −OH and −COOH groups during polycondensation and slowing down the reaction. Apparent rate constants *k* were determined from these data by fitting to a 1/(1 – *p*) = 1 + *kt* second-order kinetic law, where the conversion degree *p* = %COO*R_t_*/100 and *t* is the reaction time (Figure 3B), as previously reported for the melt polycondensation of aleuritic acid.^42^ In general, apparent *k* increased with the temperature, ranging from values close to 0 for all of the samples at 140 °C to ∼0.23 min^–1^ for AZ-100 at 200 °C. The slowing effect of zinc in the polycondensation is also evident when rate constants are compared at a specific temperature. Finally, the activation energy (*E*_a_) was calculated by the Arrhenius equation (Figure 2D). *E*_a_ decreased from ∼90.4 kJ/mol for AZ-100 (close to those reported elsewhere^42^) to ∼27.6 kJ/mol for AZ-50 (*i.e*., a reduction of ∼69%) with AZ-95 and AZ-90 with intermediate values. Such a decrease is expected since zinc carboxylates and ZnO are used to catalyze polycondensations.^45−47^ These activation energies are comparable to those of other similar polycondensation reactions to synthesize different polyesters such as poly(ethylene terephthalate) (128.4 kJ/mol) and poly(ethylene furanoate) (55.7 kJ/mol) without catalysts, poly(ethylene succinate) (59.5 kJ/mol), poly(propylene succinate) (52.0 kJ/mol), and poly(butylene succinate) (47.4 kJ/mol) catalyzed with tetrabutoxytitanium, and the resultant polyester from unsaturated and polyhydroxylated fatty acids of tomato pomace agro-wastes (28.0 kJ/mol) catalyzed with Sn(oct)_2_.^48−51^ These values of *E*_a_ are also similar to those of BPA resins: polycarbonate from BPA and diphenyl carbonate (87.9 kJ/mol) and diglycidylether of bisphenol A (60.9 kJ/mol).^52,53^

Considering the above-described data, the coatings with the highest degree of polymerization (*i.e*., those prepared at 200 °C for 60 min) were chosen for further physical characterization.

### Presence of Zinc Modifies the Physical Properties and Confers Antimicrobial Properties and Corrosion Resistance

**Figure 4:**
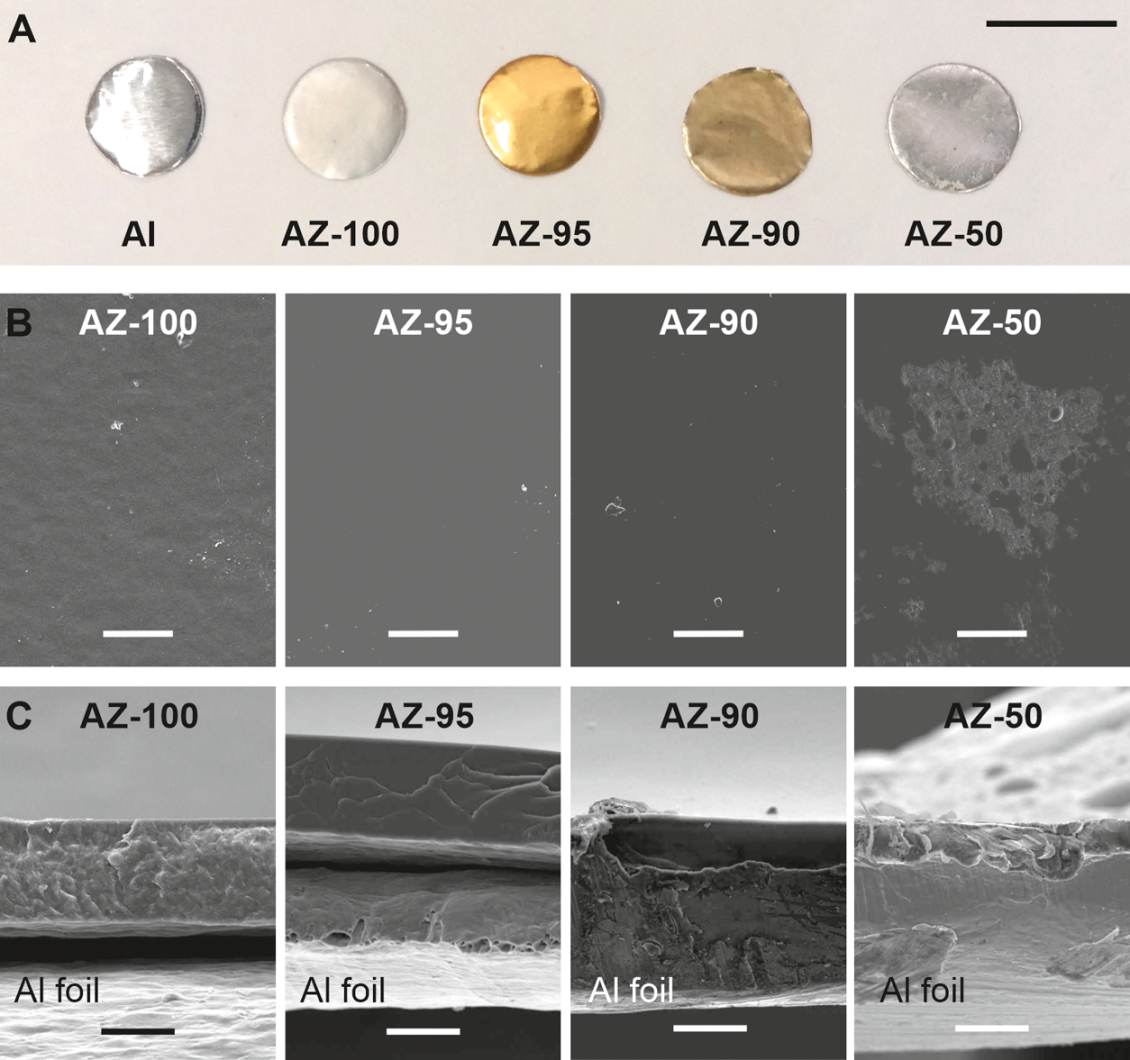
(A) Photographs of the aluminum substrate and the AZ coatings after the thermal treatment. Scale bar: 1 cm. (B, C) Top-view (scale bar: 50 μm) and cross-section (scale bar: 20 μm) SEM images, respectively, of the AZ coatings.

Figure 4A displays the photographs of the fabricated AZ samples at 200 °C for 60 min. Coatings were semitransparent with whitish (AZ-100), goldish (AZ-95), and brownish (AZ-90) colors. The macroscopic appearance of AZ-100 is similar to those previously described for other samples of pure polyaleuritate on metal substrates.^54^ The goldish and brownish colors can be attributed to the presence of zinc carboxylates. For instance, zinc carboxylates from unsaturated fatty acids show a yellow color.^44^ AZ-50 showed a whitish and less translucent surface compared to the others. Such appearance could result from the partial and inhomogeneous covering of the aluminum substrate, as reported in Figure 4B, and the consequent increase of the scattered light.

The micromorphology of the zinc polyaleuritate ionomers was characterized by SEM (Figure 4B,C). In particular, Figure 4B displays the surface of AZ coatings. For AZ-100, AZ-95, and AZ-90, a uniform and homogeneous topography is observed. Instead, the AZ-50 sample showed a rough surface with few areas not completely covered with polyaleuritate. This is due to the low amount of sprayed aleuritic acid on the ZnO layer during the coating fabrication. The cross-sections of the ionomers were also analyzed (Figure 4C). Coatings were uniform, with exception of AZ-50, and did not present fractures. The thickness depended directly on the amount of sprayed aleuritic acid and varied from ∼25 μm for AZ-100 to ∼10 μm (maximum thickness for covered areas) for AZ-50.

**Figure 5:**
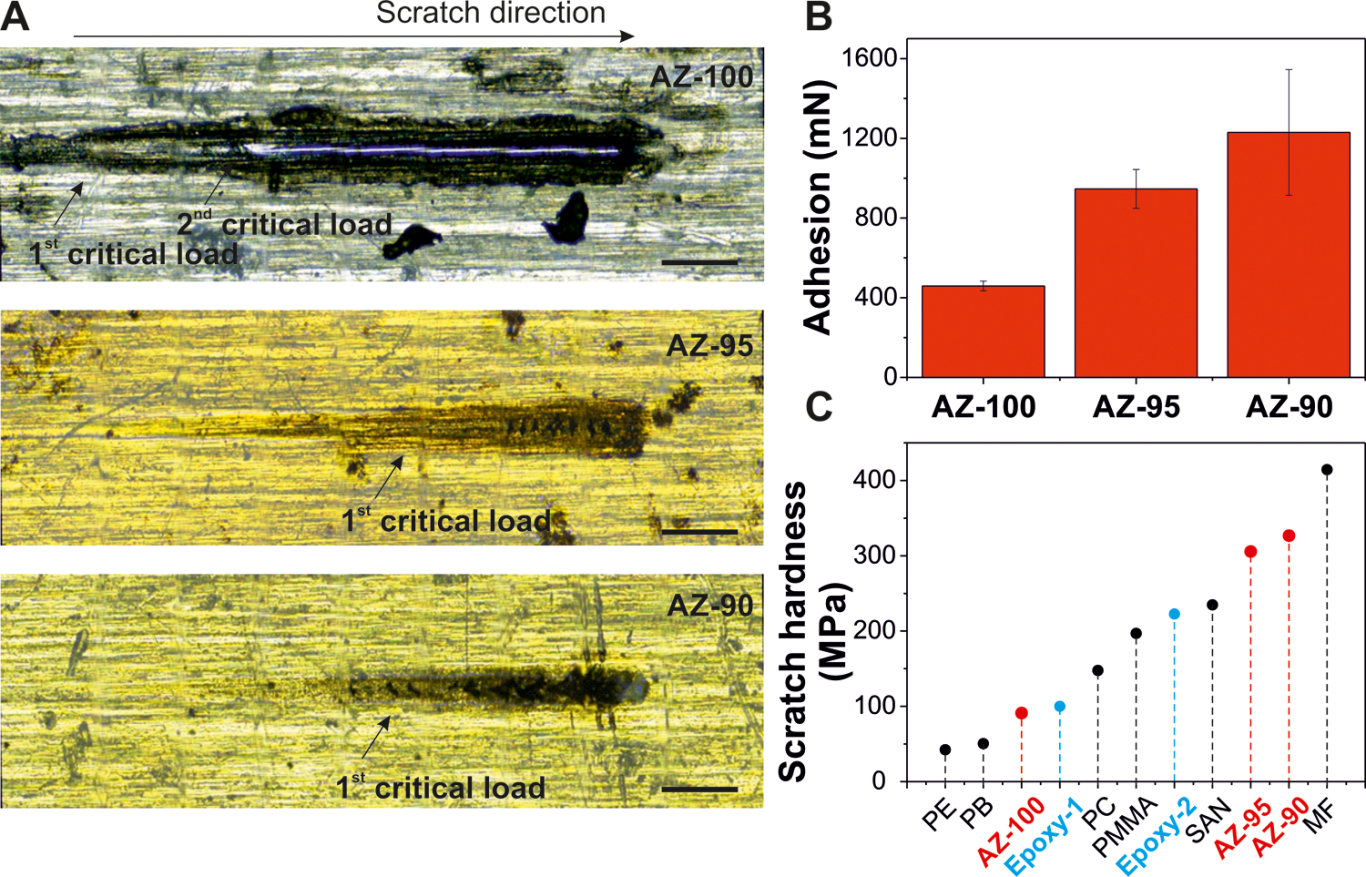
(A) Typical scratch scars for AZ-100, AZ-95, and AZ-90 samples. The scratch direction and the onsets of the first (first damage mechanism or plunging) and second (second damage or substrate exposure) critical loads are indicated. Scale bar: 0.5 mm. (B) Adhesion of AZ-100, AZ-95, and AZ-90 coatings. (C) Scratch hardness of AZ-100, AZ-95, and AZ-90 compared to those of common polymers used in coatings.

Progressive load scratch tests were performed to evaluate the adhesion to aluminum plates of AZ coatings prepared at 200 °C for 60 min. The typical scratches and the average values of the critical load are reported in Figure 5. For all coatings, the damage mechanism was that of a semirigid polymer: the tip slowly penetrates into the material until the coating is displaced sideways. The onset of damage, characterized by a widening halo around the scratch line, is taken as the first critical load, while the second one is defined as the one for which the tip reaches the substrate, as indicated in Figure 5A. The first and second critical loads started at (340 ± 180) mN and (530 ± 170) mN, respectively, for the AZ-100 sample. The AZ-90 and AZ-95 coatings showed similar first damage mechanisms at much higher loads (Figure 5B). Both did not present any damage within the initial load range. For this reason, additional tests were conducted up to 2 N maximum load. The first critical load was found at (946 ± 98) and (1140 ± 360) mN for AZ-95 and AZ-90, respectively. The second damage mechanism was not detected in these samples for the load range tested, as observed in Figure 5A. For the AZ-50 coating, the detection of critical loads was severely affected by the substrate’s incomplete coverage and the consequent specimen roughness. For this reason, scratch tests were discarded in this sample. It should be pointed out that both kinds of damage are cohesive and localized in the coating. No loss of adhesion (delamination and chipping) was observed in any material tested. The increase of adhesion induced by Zn has been described, which is commercially exploited in analogous structures such as zinc polycarboxylates (mainly polyacrylates), usually known as dental luting cement.^55^ In fact, the adhesion of these zinc polycarboxylates to aluminum-based surfaces has been reported as very high.^56^ Scratch hardness tests (Figures 5C and S3) showed similar results: the AZ-100 sample exhibited the worst performance with a value of ∼91 MPa, while AZ-95 and AZ-90 had similar behavior with values of ∼306 and ∼327 MPa, respectively (*i.e*., an increase of ∼236 and ∼259%, respectively). Such reinforcement of mechanical properties produced by different zinc carboxylates has been previously reported for several rubbers, elastomers, and vulcanizate thermoplastics.^57−60^ Finally, the AZ values of scratch hardness were compared to those of other manmade polymers and resins: polyethylene (PE), polybutene (PB), two different types of bisphenol A-based epoxy resins (Duroglass P5/1 labeled as Epoxy-1 and Ampreg 26 labeled as Epoxy-2), polycarbonate (PC), poly(methyl methacrylate) (PMMA), styrene-acrylonitrile copolymer (SAN), and melamine formaldehyde (MF).^61^ As observed, AZ-100 shows similar values to soft polymers such as PB and Epoxy-1, while AZ-90 and AZ-95 are comparable to very robust SAN and MF, overcoming the performances of typical epoxy resins. It is important to mention that for this comparison, values from neat polymers with no fillers have been used. The incorporation of particles into polymer matrices can effectively improve their scratch hardness.

**Figure 6:**
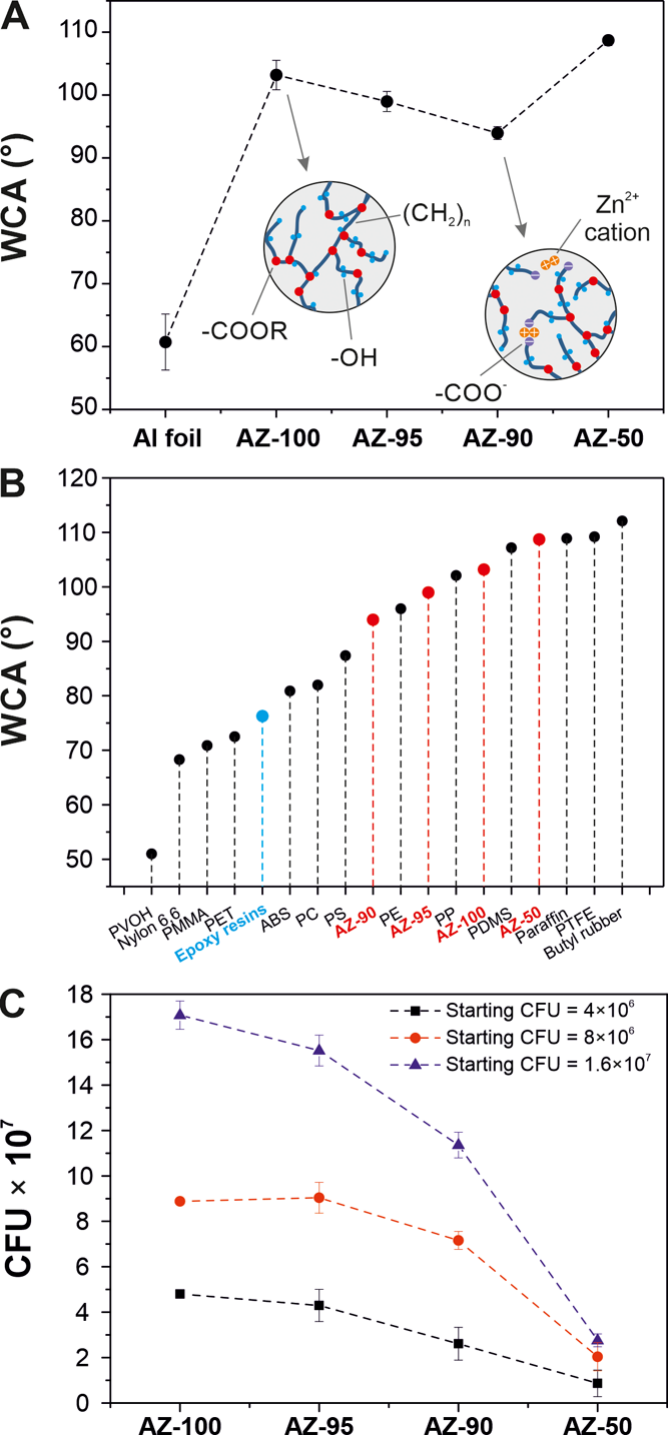
(A) Water contact angles of the aluminum substrate and AZ-100, AZ-95, AZ-90, and AZ-50 coatings. Insets show schematic models at the molecular level of pure polyaleuritate (AZ-100) and zinc-containing samples. Main chemical species are indicated. (B) Comparison of WCA values of AZ coatings to those of common petroleum-based plastics. (C) *E. coli* growth on AZ coatings at three different starting bacteria concentrations as an indicator of antimicrobial activity.

Water contact angles (WCAs) of the different AZ samples fabricated at 200 °C for 60 min were determined to evaluate the wettability and are shown in Figure 6A. The value for the Al substrate was ∼61°, typical of hydrophilic materials. The WCA of AZ-100 was increased to ∼103°, that is, a hydrophobic behavior. AZ-95 and AZ-90 ionomers exhibited lower values (∼99 and ∼94°, respectively) than the coating made of pure polyaleuritate. Most likely, the smoother surfaces, as observed by top-view SEM images, and the participation of polar charged species (*viz*., Zn^2+^ and −COO^–^ groups, as indicated in the insets of Figure 6A) that can interact with water molecules are suggested to contribute to this decrease. AZ-50 showed the highest WCA with a value of ∼109°. This behavior can be associated with the roughness induced by the incomplete coverage of the surface. These values were compared to those of other manmade polymers: poly(vinyl alcohol) (PVOH), nylon 66, PMMA, poly(ethylene terephthalate) (PET), a typical epoxy resin, acrylonitrile butadiene styrene (ABS), PC, polystyrene (PS), PE, polypropylene (PP), poly(dimethylsiloxane) (PDMS), paraffin, poly(tetrafluoroethylene) (PTFE), and a butyl resin.^51,62^ As observed, AZ coatings showed hydrophobicity similar to common plastics, such as PE and PP, and elastomers, such as PDMS, and much higher than typical epoxy resins (WCA ∼ 76°).

The antimicrobial activity of zinc polyaleuritate ionomers against *E. coli* was also investigated. *E. coli* is a common pathogenic bacterium that can be found in canned food and cause foodborne illness outbreaks, even in developed countries, producing critical economic losses and health problems to humans and animals.^63,64^ The growth of *E. coli*, quantified by the number of colony forming units (CFU) from three different starting populations, on the AZ coatings is reported in Figure 6C. In general, the presence of zinc reduced the number of CFU with a better effect as the proportion of the metal was higher. The sample with the highest concentration of zinc (AZ-50) displayed a decrease in CFU of ∼80%, for all starting concentrations of bacteria. Samples with lower concentrations of the metal, namely, AZ-95 and AZ-90, still displayed antibacterial effects compared to control AZ-100 but with lower efficacy, reducing bacterial growth by ∼10 and ∼30%, respectively. These results were expected since Zn^2+^ (in ZnO or as a carboxylate) can damage the bacterial cell membrane and produce bacteria’s death.^65^ In fact, ZnO nanoparticles and zinc polyester-based ionomers have been used as antimicrobial agents against *E. coli*.^66,67^

**Figure 7:**
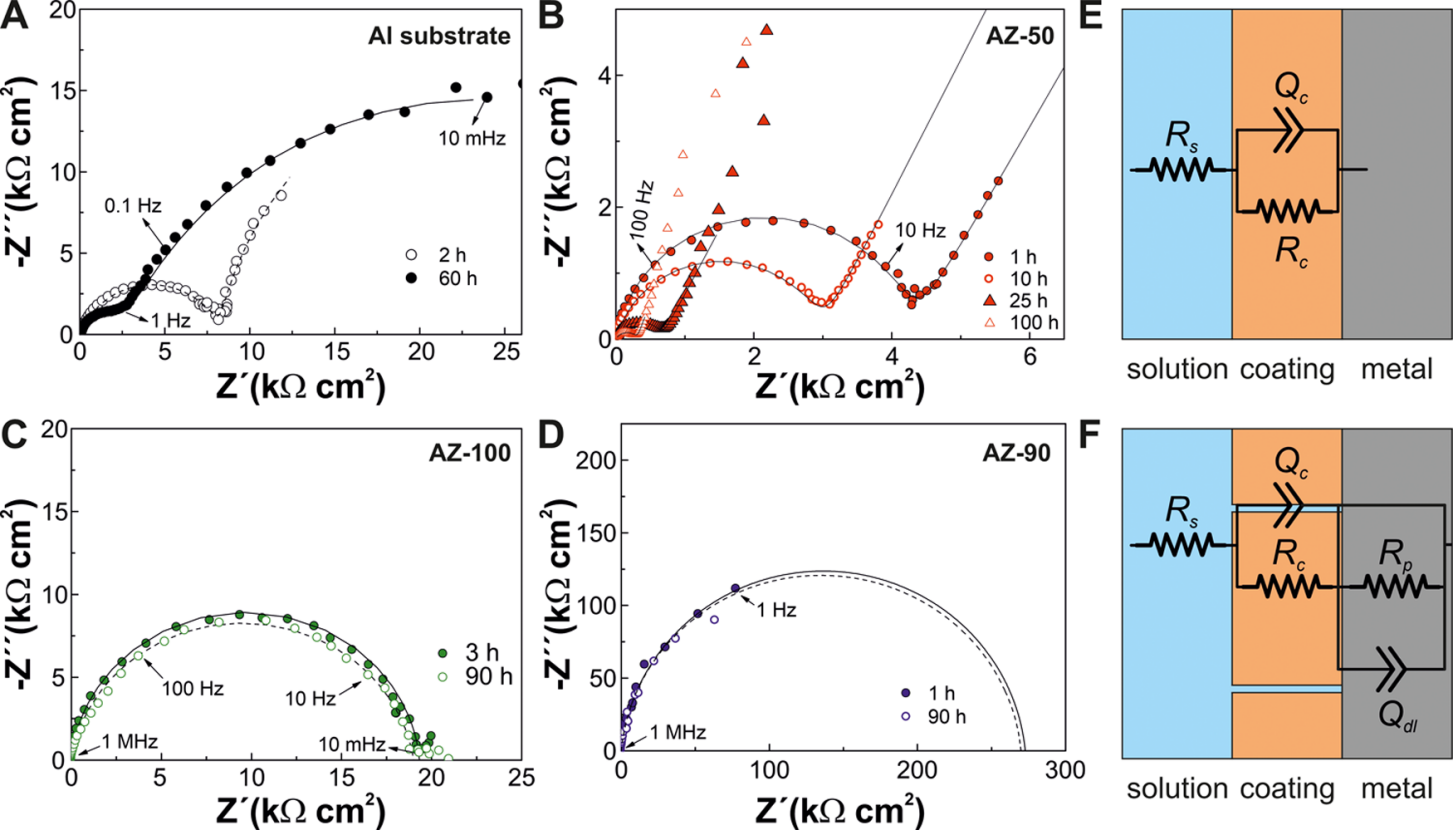
(A–D) Nyquist plots for the aluminum substrate, AZ-50, AZ-100, and AZ-90, respectively. (E, F) Equivalent circuit models for an intact coating and a porous coating, respectively. The fitting curves obtained by equivalent circuits are represented by lines.

Figure 7A–D shows the Nyquist plots of the coatings at different immersion times. The uncoated substrate exhibited a single semicircle at the beginning of the durability test, which is assigned to the charge transfer reaction during the corrosion process at the metal/solution interface (Figure 7A). A second contribution that resembled an incomplete semicircle appeared after several hours of immersion at a low-frequency range, which is attributed to the diffusion of ions through the formed corrosion layer.^68^ In the case of the AZ-50 coating, a broad depressed semicircle is observed in the Nyquist plots with a resistance, determined from the diameter of the semicircle, of 4 MΩ cm^2^ (Figure 7B). However, the resistance of this contribution decreased over time due to severe coating damage as a consequence of the incomplete substrate coverage of AZ-50, as previously observed by SEM. The AZ-100, AZ-90, and AZ-95 coatings presented only a large semicircle in the Nyquist plot that remained unchanged during the immersion, indicating no coating delamination (Figure 7C,D). The Nyquist plots of AZ-95 were similar to those of AZ-90, and for simplicity, they were not included here. It is also worth noting that the resistance of AZ-95 was 1 order of magnitude superior to AZ-100, suggesting that Zn addition improves the protective performance of these coatings. In fact, zinc aliphatic carboxylates and ZnO nanoparticles are used as corrosion resistance agents.^69−71^

The Nyquist plots were analyzed using appropriate equivalent circuits to obtain further insights into the electrochemical processes occurring at the coating/metal interface.^68,72^ The equivalent circuits used for the different coatings are displayed in Figure 7E,F. For the stable coatings, the equivalent circuit comprises resistance *R*_s_, assigned to the solution, in serial with an (*R*_c_*Q*_c_) element, where *R*_c_ is the coating resistance in parallel with a constant phase element *Q*_c_ with impedance *Z*_Q_ = *Q*_o_(*jw*)^−*n*^, where *w* is the angular frequency, *Q*_o_ is the pseudocapacitance, and *n* is an exponential coefficient to consider the nonideal capacitive behavior. The real coating capacitance *C*_c_ was determined using the following relation^73^
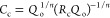
4The unprotected substrate and the AZ-50 coating required an alternative equivalent circuit to account for the two electrochemical interfaces that exist between the electrolyte solution and the metal interface (Figure 7F). An additional *R*_p_–*Q*_dl_ element was needed to describe the change transfer processes at the coating/metal interface, where *R*_p_ is the electrode polarization resistance and *Q*_dl_ is the double-layer capacitance.^68^

The solution resistance had a value of 96 Ω cm^2^ and was nearly constant over immersion time, suggesting the minimal presence of produced corrosion ions into the solution for the different coatings. Regarding the coating resistance, it remained almost invariable over immersion time for AZ-90, AZ-95, and AZ-100 (Figure S4A). In contrast, *R*_c_ decreased for AZ-50 due to water penetration into the coating during the corrosion process. Moreover, the capacitances of AZ-95 and AZ-90 were nearly constant during the durability test with a value of 120 pF cm^2^, while slightly increased from 120 to 300 pF/cm^2^ for AZ-100, indicating some electrolyte diffusion through the coating (Figure S4B). Overall, the degrees of protection offered by both AZ-90 and AZ-95 were nearly equivalent as both coatings have reached an impedance module above 10 MΩ cm^2^, a value required for adequate level protection against corrosion.

### Migration of Zinc and Polyaleuritate to the Food Simulant is below of Regulated Limits

**Figure 8:**
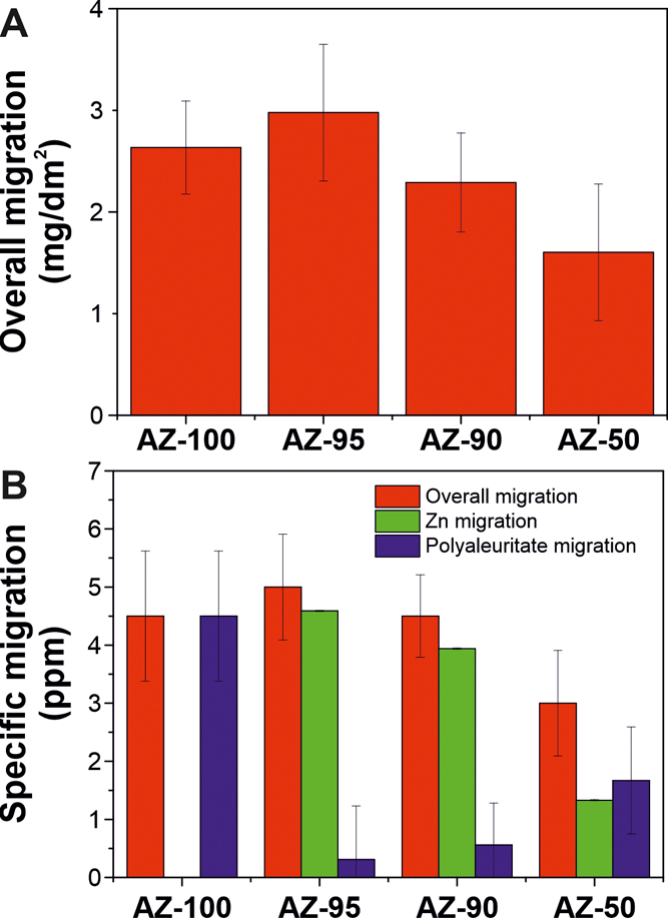
(A) Overall migration and (B) comparison of the overall, zinc, and polyaleuritate migrations of AZ coatings in a 10% (v/v) ethanol solution.

To check the possibility of using these coatings in food packaging, migration tests were carried out as indicated by the European legislation (Figure 8).^74^ For this, the denominated food simulant A, a 10% (v/v) ethanol solution, was used, although the analysis with other simulants (*e.g*., ethanol 20 and 50%, acetic acid 3%, vegetable oil, and solid particles of poly(2,6-diphenyl-*p*-phenylene oxide)) is necessary to check the universality of zinc polyaleuritate ionomers. This food simulant is assigned to hydrophilic food and can extract hydrophilic substances. There are several examples in this category: molasses; sugar syrups; honey; sauces; mustard; nuts in the paste or cream form; and vegetables, fishes, meats, and shells (fresh or in an oily medium), among others. Figure 8A displays the overall migration levels for all AZ coatings prepared at 200 °C for 60 min. The values are ranged between ∼3.0 and ∼1.6 mg/dm^2^, well below the limit of 10 mg/dm^2^ specified in the European regulation for plastics in contact with food. As an indication, the aluminum neat film migration level was ∼3.0 mg/dm^2^. The specific migration of zinc and polyaleuritate (that can also include unreacted aleuritic acid monomers) was also investigated (Figure 8B). In all cases, the specific migration of zinc was <5 ppm, very far from the legislated migration limit of 25 ppm (or 25 mg per kg of food or food simulant) for this metal. The specific migration of polyaleuritate was maximum for AZ-100 with a value of ∼4.5 ppm. The specific value was below 2 ppm for zinc-containing coatings. These low values of overall and specific migrations can be explained by the thermoset characteristics of polyaleuritate (*i.e*., insolubility)^21^ and the high adherence of AZ coatings to aluminum substrates. Although there are no reports about aleuritic acid toxicity when used as a food additive, the maximum dose for shellac (mainly composed of aleuritate units) to avoid toxic effects is 5000 ppm.^75^

## Conclusions

Biorenewable aleuritic acid and ZnO were used to fabricate sustainable bisphenol A-free lacquers for metal packaging. Zinc polyaleuritate ionomer coatings were prepared using a two-step procedure: first, ZnO NPs were obtained by thermal decomposition of zinc acetate, and then aleuritic acid was sprayed and polymerized by melt polycondensation. Time (15, 30, 45, and 60 min), temperature (140, 160, 180, and 200 °C), and relative amounts of ZnO/aleuritic acid (0:100, 5:95, 10:90, and 50:50, w/w) were investigated to optimize the process. In such conditions, both components react to form zinc polyaleuritate ionomers. Time and temperature accelerate the reaction, while zinc presence slows down the polycondensation and decreases the activation energy. The color, morphology, adhesion, hardness, wettability, and antibacterial properties depended on the zinc amount. Both adhesion to Al substrate and hardness were significantly increased, while the wettability was typical of hydrophobic manmade resins and plastics, surpassing BPA resins’ characteristic values. The presence of zinc confers a certain degree of antibacterial properties to the coatings. Electrochemical impedance spectroscopy revealed that Zn-containing coatings AZ-90 and AZ-95 exhibit better protective barrier properties against corrosion. All lacquers show values of overall and specific migrations well below the regulated European standards. In view of the above-described properties, these zinc polyaleuritate ionomer lacquers can be realistic and sustainable alternatives to BPA-based resins in metal packaging.

## Supporting Information Available

XRD diffractogram of the bare Al foil and Al/zinc acetate sample before the thermal treatment; HR-SEM top-view image of the ZnO layer, fully covering the underneath aluminum substrate; particle size distribution of the synthesized ZnO NPs; electron diffraction (SEAD) patterns of the ZnO NPs observed by TEM; AFM height profile of the ZnO nanoparticles covering the aluminum substrate; contribution to the C=O stretching mode of carboxylate, carboxyl, and total ester groups of AZ-100, AZ-95, AZ-90, and AZ-50 at 140, 160, 180, and 200; scratch hardness scars of AZ-100, AZ-95, and AZ-90; and coating resistance and capacitance over time (PDF)
