# Supplementary material for: Zinc Polyaleuritate Ionomer Coatings as a Sustainable, Alternative Technology for Bisphenol A-Free Metal Packaging
Source: ACS Sustain Chem Eng. 2021 Nov 3;9(46):15484–95. doi: 10.1021/acssuschemeng.1c04815 (PMC8611806; doi:10.1021/acssuschemeng.1c04815)
Supplement: Supplementary file 1 — sc1c04815_si_001.pdf [file sc1c04815_si_001.pdf]

SUPPLEMENTARY INFORMATION

Zinc polyaleuritate ionomer coatings as a sustainable, alternative technology for bisphenol A-free metal packaging

Davide Morselli[1,2], Pietro Cataldi[1,3], Uttam Chandra Paul[1], Luca Ceseracciu[4], Jose Jesus Benitez[5], Alice Scarpellini[6], Susana Guzman-Puyol[1,7], Antonio Heredia[8], Paola Valentini[9], Pier Paolo Pompa[9], David Marrero-López[10], Athanassia Athanassiou[1], and José Alejandro Heredia-Guerrero[1,7]**

[1] Smart Materials, Istituto Italiano di Tecnologia, Via Morego 30, 16163 Genova, Italy.

[2] Department of Civil, Chemical, Environmental and Materials Engineering (DICAM), Università di Bologna, Via Terracini 28, 40131 Bologna, Italy.

[3] Center for Nano Science and Technology @PoliMi, Istituto Italiano di Tecnologia, Via G. Pascoli 70/3, 20133 Milan, Italy

[4] Materials Characterization Facility, Istituto Italiano di Tecnologia, Via Morego 30, 16163 Genova, Italy.

[5] Instituto de Ciencia de Materiales de Sevilla, Centro mixto CSIC-Universidad de Sevilla, Americo Vespucio 49, Isla de la Cartuja, Sevilla, 41092, Spain.

[6] Electron Microscopy Facility, Istituto Italiano di Tecnologia, Via Morego, 30, Genova, 16163, Italy.

[7] Instituto de Hortofruticultura Subtropical y Mediterránea "La Mayora", Universidad de Málaga-Consejo Superior de Investigaciones Científicas (IHSM, UMA-CSIC), Bulevar Louis Pasteur, 49, 29010 Málaga, Spain.

[8] Instituto de Hortofruticultura Subtropical y Mediterránea "La Mayora", Universidad de Málaga-Consejo Superior de Investigaciones Científicas (IHSM, UMA-CSIC), Departamento de Biología Molecular y Bioquímica, Facultad de Ciencias, E-29071 Málaga, Spain.

[9] Nanobiointeractions & Nanodiagnostic, Istituto Italiano di Tecnologia, Via Morego 30, 16163 Genova, Italy.

[10] Universidad de Málaga, Dpto. de Física Aplicada I, 29071-Málaga, Spain.

*Corresponding author: davide.morselli6@unibo.it.

**Corresponding author: ja.heredia@csic.es. Number of pages: 5.

Number of figures: 4.

Number of tables: 0.

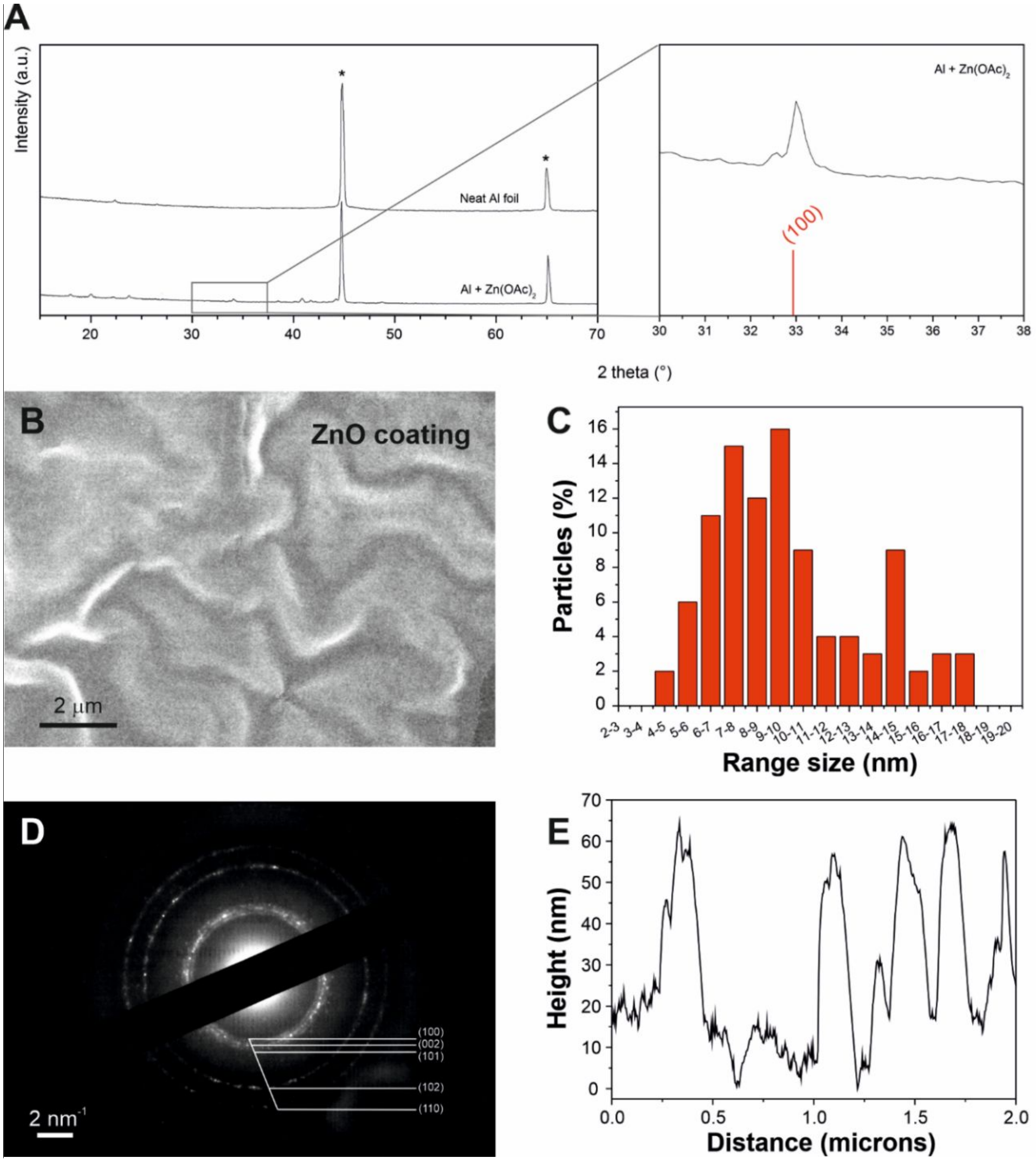

Figure S1. A, XRD diffractogram of the bare Al foil and Al/zinc acetate sample before the thermal treatment. (*) indicates the reflections due to the aluminum foil used as substrate. On the right, the magnification of the range 30-38° (2θ) and the associated zinc acetate database pattern (JCPDS file 00-056-0569, in red) are shown. **B**, HR-SEM top-view image of the ZnO layer, fully covering the underneath aluminum substrate. **C**, particle size distribution of the synthesized ZnO NPs. **D**, electron diffraction (SEAD) patterns of the ZnO NPs observed by TEM. The assignment of main crystalline planes are included **E**, AFM height profile of the ZnO nanoparticles covering the aluminum substrate.

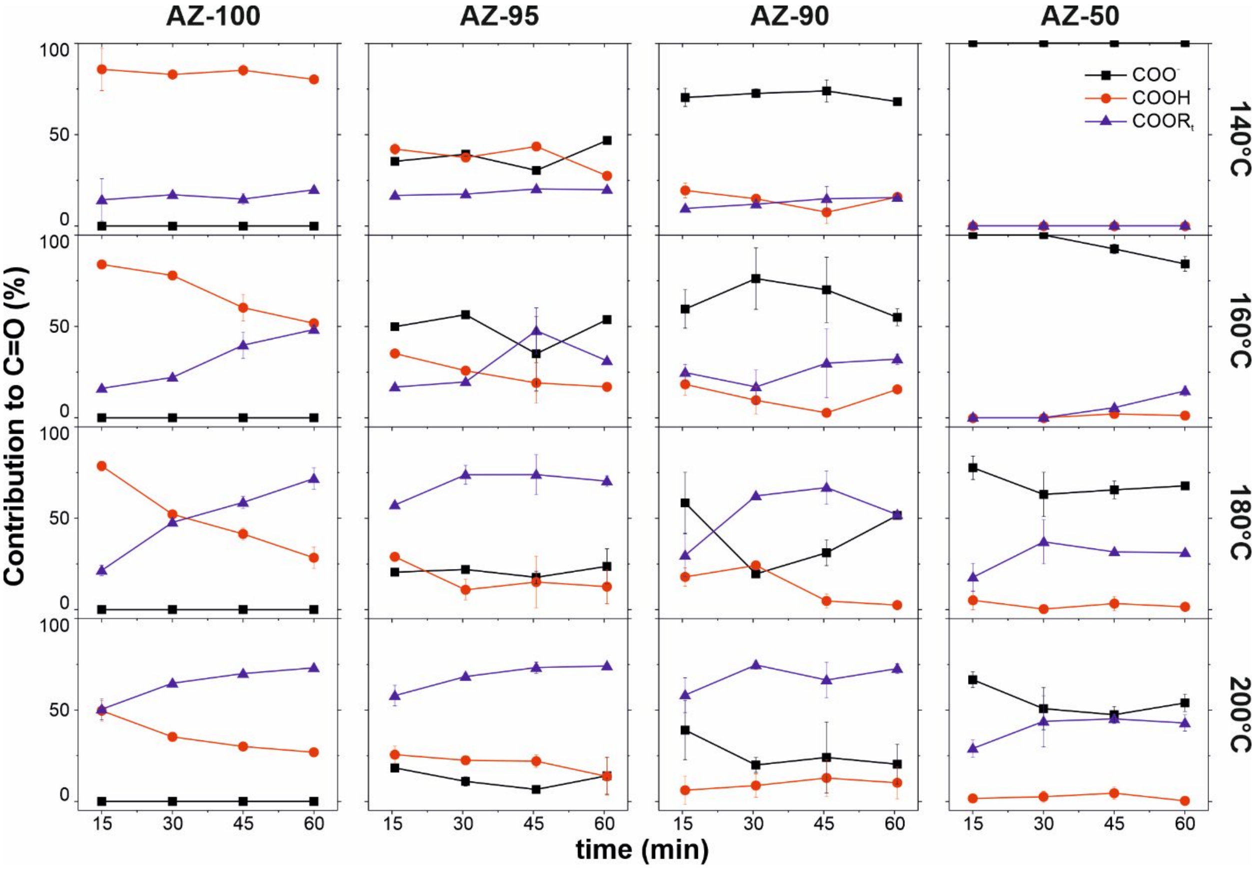

Figure S2. Contribution to the C=O stretching mode of carboxylate, carboxyl, and total ester groups of AZ-100 (first column), AZ-95 (second column), AZ-90 (third column), and AZ-50 (fourth column) at 140 (first row), 160 (second row), 180 (third row), and 200 (fourth row).

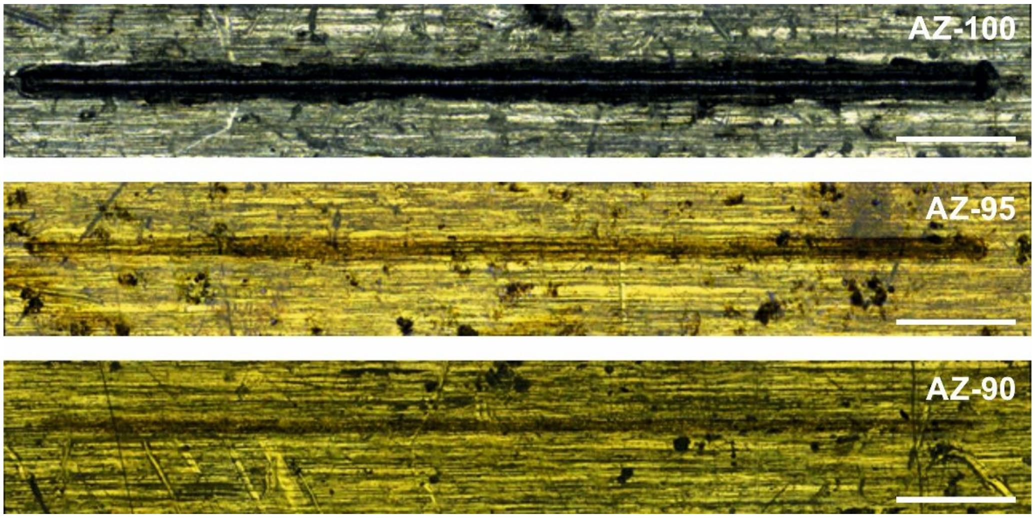

Figure S3. Scratch hardness scars of AZ-100, AZ-95, and AZ-90. Scale bar: 625 μm.

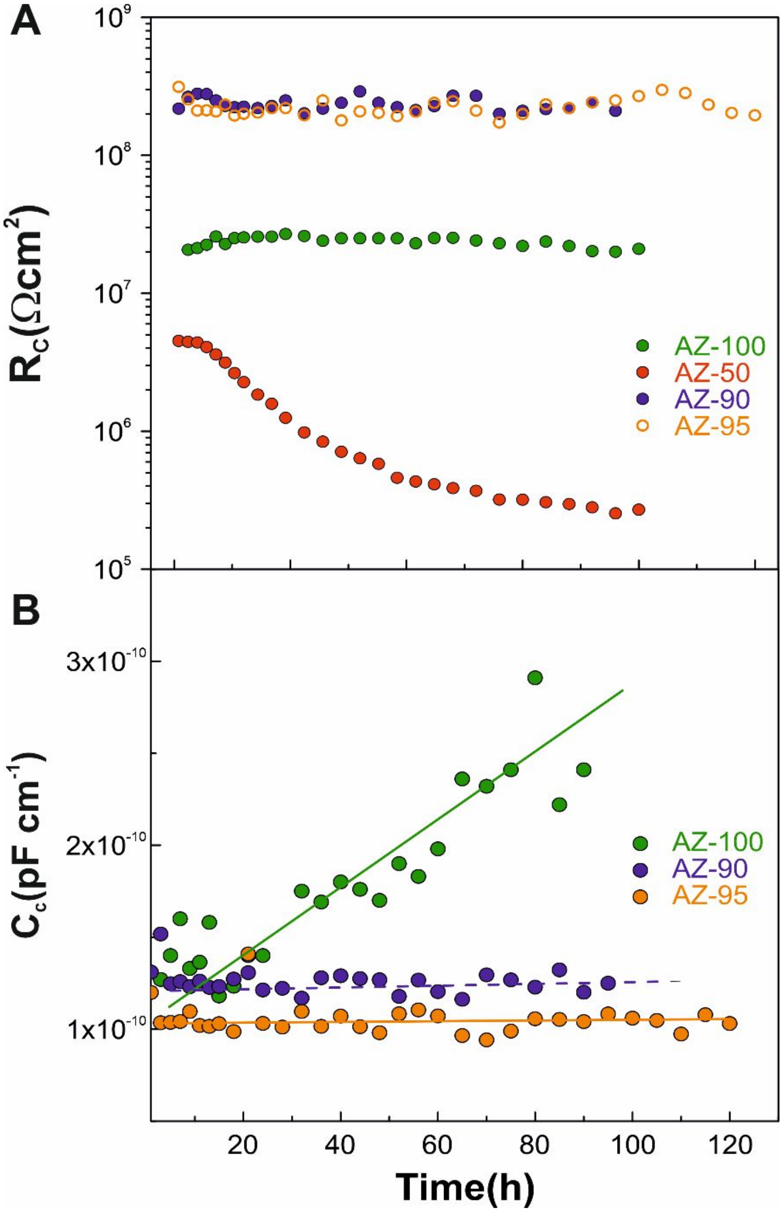

Fig. S4. A, **B**, variation of coating resistance and coating capacitance, respectively, over time.
